# Comparable outcomes in patients with B-cell acute lymphoblastic leukemia receiving haploidentical hematopoietic stem cell transplantation: Pretransplant minimal residual disease-negative complete remission following chimeric antigen receptor T-cell therapy versus chemotherapy

**DOI:** 10.3389/fimmu.2022.934442

**Published:** 2022-08-30

**Authors:** Ting-Ting Yang, Ye Meng, De-Lin Kong, Guo-Qing Wei, Ming-Ming Zhang, Wen-Jun Wu, Ji-Min Shi, Yi Luo, Yan-Min Zhao, Jian Yu, Rui-Rui Jing, Meng-Yu Zhao, Hou-Li Zhao, He Huang, Yong-Xian Hu

**Affiliations:** ^1^ Bone Marrow Transplantation Center, The First Affiliated Hospital, Zhejiang University School of Medicine, Hangzhou, China; ^2^ Liangzhu Laboratory, Zhejiang University Medical Center, Hangzhou, China; ^3^ Institute of Hematology, Zhejiang University, Hangzhou, China; ^4^ Zhejiang Province Engineering Laboratory for Stem Cell and Immunity Therapy, Hangzhou, China

**Keywords:** MRD-negative CR, chimeric antigen receptor (CAR)-T, haploidentical HSCT, first complete remission, acute lymphoblastic leukemia

## Abstract

**Introduction:**

Chimeric antigen receptor (CAR) T-cell (CAR-T) therapy followed by haploidentical hematopoietic stem cell transplantation (haplo-HSCT) markedly improves the long-term survival of patients with refractory/relapsed (R/R) B-cell acute lymphoblastic leukemia (B-ALL).

**Methods:**

We performed a parallel comparison of transplant outcomes in 168 B-ALL patients undergoing haplo-HSCT after achieving minimal residual disease (MRD)-negative complete remission (CR) from CAR-T therapy (n = 28) or chemotherapy (n = 140) between January 2016 and August 2021. We further divided the chemotherapy group into the first CR group (chemo+CR1, n = 118) and a second or more CR group (chemo+≥CR2, n = 22).

**Results:**

With a median follow-up period of 31.0 months, the 2-year overall survival (OS), leukemia-free survival (LFS), non-relapse mortality (NRM), and relapse rates in the CAR-T and chemotherapy groups did not differ significantly (OS, 87.9% vs. 71.5 %; LFS, 72.0% vs. 66.8%; NRM, 3.9% vs. 13.7%; relapse, 24.1% vs. 19.4%). Multivariate analysis confirmed that ≥CR2 at transplantation following chemotherapy was an independent risk factor associated with poor OS (hazard ratio (HR) 4.22 [95% CI, 1.34–13.293], *p* = 0.014) and LFS (HR 2.57 [95% CI, 1.041–6.343], *p* = 0.041). The probabilities of OS and LFS at 2 years in the CAR-T group were comparable to those in the chemo+CR1 group but significantly higher than those in the chemo+≥CR2 group (OS, 87.9% vs. 37.8%, *p* = 0.007; LFS, 72.0% vs. 41.7%, *p* = 0.043). No significant differences in the incidences of NRM were noted among the three groups.

**Conclusions:**

Our results demonstrated that patients with R/R B-ALL receiving haplo-HSCT after CAR-T therapy achieved comparable outcomes to patients transplanted post-chemotherapy-based MRD-negative CR1, without increased risk of transplant-related mortality and toxicity.

## Introduction

Adult patients diagnosed with refractory/relapsed (R/R) B-cell acute lymphoblastic leukemia (B-ALL) have a poor prognosis, with a 5-year overall survival (OS) rate of 10%–20% (1, 2). Allogeneic hematopoietic stem cell transplantation (allo-HSCT) is recommended as the frontline treatment option for R/R B-ALL. However, the prognosis of high-risk patients with R/R B-ALL or those positive for minimal residual disease (MRD) pre-allo-HSCT remains dismal (3, 4). MRD negativity at the time of allo-HSCT significantly correlates with post-transplant long-term survival and a lower risk of relapse (5–9). Salvage chemotherapy pre-allo-HSCT for R/R ALL patients is usually accompanied by serious complications and chemoresistance, leading to an unsatisfactory response rate of 31%–44% after the first salvage chemotherapy and 18%–25% after the second salvage chemotherapy (1, 3, 4, 10–12). Thus, novel therapeutic approaches that can effectively achieve complete remission (CR) and can decrease MRD before HSCT are urgently required.

In recent years, cellular immunotherapy of CD19-directed chimeric antigen receptor (CAR) T cells (CAR-T) has shown impressive therapeutic responses in R/R B-ALL patients, with high CR rates ranging from 70% to 94%, dramatically altering the treatment strategy for R/R ALL (13–17). In addition, CAR-T therapy has shown superior efficacy in eradicating MRD. Previous large-scale clinical trials found that the MRD-negative CR (MRD**^−^** CR) rate following CAR-T therapy is approximately 60% to 90%, which is higher than that induced by conventional chemotherapy (13, 15–21). In our previous study, encouraging CR was achieved in R/R B-ALL patients receiving CAR-T therapy, with an MRD**^−^** CR rate of 92.3% at 1 month after CAR-T therapy (22). Nevertheless, 30%–50% of patients eventually relapsed after CAR-T therapy (16). Emerging studies have suggested that patients at high risk of relapse would benefit from consolidative allo-HSCT following CAR-T therapy (13, 14, 23, 24). Hay et al. observed improved OS (72%) and leukemia-free survival (LFS) (61%) at 2 years in R/R patients receiving consolidative allo-HSCT in MRD**^−^** CR induced by CAR-T therapy, with a lower 2-year cumulative incidence of relapse (CIR) of 17% (24). These outcomes are comparable to those previously reported by Cassaday et al., with OS, LFS, and CIR in patients transplanted in MRD-negative first CR (CR1) following chemotherapy of approximately 72%, 62%, and 19%, respectively (25). Nevertheless, studies on the direct comparison of efficacy and safety of transplant after achieving MRD**^−^** CR following CAR-T therapy versus chemotherapy are limited.

In the present study, we aimed to analyze transplantation outcomes after haploidentical HSCT (haplo-HSCT) in R/R patients with MRD**^−^** CR from CAR-T therapy, choosing patients who received haplo-HSCT after MRD**^−^** CR post-chemotherapy over the same period as a control group.

## Materials and methods

### Study population

A total of 168 consecutive B-ALL patients who underwent haplo-HSCT after achieving MRD-negative CR either from chemotherapy or from CAR-T therapy at our center between January 2016 and August 2021 were enrolled. B-ALL patients with MRD-positive or MRD-unknown at transplant, not in remission, or receiving matched sibling donor transplantation or unrelated transplantation were excluded. Based on the treatment procedures prior to HSCT, 140 patients were classified into the chemotherapy group, and 28 patients were assigned to the CAR-T group. The chemotherapy patients were then subgrouped in terms of remission status before haplo-HSCT: patients in CR1 who achieved MRD negativity followed by haplo-HSCT (chemo+CR1 group, n = 118) and patients in second or more CR who achieved MRD negativity followed by HSCT (chemo+≥CR2 group, n = 22). These two cohorts were compared separately to the CAR-T group. All patients enrolled in our study were from clinical trials (www.clinicaltrials.gov as #NCT04532268 and #NCT04532281; www.chictr.org.cn as #ChiCTR-OCC-15007008 and #ChiCTR-ORN-16008948). The study protocol was approved by the Institutional Review Board of the First Affiliated Hospital, School of Medicine, Zhejiang University. Written informed consent was obtained from all the patients in accordance with the Declaration of Helsinki.

### CD19-directed chimeric antigen receptor T-cell therapy

The manufacture of CAR-T and details of the treatment protocol have been described in previous studies (22, 26). Anti-CD19 chimeric antigen receptor T cells constructed with a 4-1BB costimulatory domain were generated using the lentiviral vector from fresh leukapheresis material by Shanghai YaKe Biotech Company (Shanghai, China). Before CAR-T infusion, all CAR-T required quality control according to the code of manufacturing quality management for chimeric antigen receptor T cell-based medicinal products formulated by the China Medicinal Biotech Association. The lymphodepleting chemotherapy regimen fludarabine included 30 mg/m^2^ on days −4 to −2 and cyclophosphamide 500 mg/m^2^ on days −3 to −2, followed by infusion (day 0) of anti-CD19 CAR-T.

### Haploidentical hematopoietic stem cell transplantation

All the patients underwent haplo-HSCT with peripheral blood stem cells from haploidentical donors. The conditioning regimen included either myeloablative conditioning (MAC) or reduced-intensity conditioning (RIC) regimens based on patient age, performance status, comorbidities, and prior treatment strategies. The myeloablative conditioning regimen consisted of cytarabine (4 g/m^2^/day i.v. on days −10 to −9), busulfan (3.2 mg/kg/day i.v. on days −8 to −6), cyclophosphamide (1.8 g/m^2^/day i.v. on days −5 to −4), Me-CCNU 250 mg/m^2^ orally on day −3, and rabbit anti-thymocyte globulin (ATG; thymoglobulin; Sanofi, Paris, France) (1.5 mg/kg/day i.v. on days −5 to −2). The other eight patients received a Flu-Bu-ATG-based regimen (fludarabine 30 mg/m2/day i.v. on days −10 to −5, busulfan 3.2 mg/kg/day i.v. on days −6 to −5, and ATG 5 mg/kg/day i.v. on days −4 to −1) for RIC. At least 4 × 10^8^ mononuclear cells (MNCs) and 2 × 10^6^ CD34+ cells per kilogram of recipient body weight were expected to be collected.

Graft-versus-host disease (GVHD) prophylaxis, including cyclosporin A (CSA) or tacrolimus (FK506), short-term methotrexate (MTX), and mycophenolate mofetil (MMF), was administered to all patients. CSA or FK506 was initiated intravenously at 2.5 mg/kg/day starting on day −7, and the dose was adjusted to maintain the target blood concentration of 200 to 300 ng/ml for CSA and of 5 to 10 ng/ml for FK506. Based on the chimeric status and evidence of GVHD, the dose of CSA or FK506 was gradually tapered during the second month of post-transplantation, ending in complete withdrawal during the ninth month after haploidentical related donor (HRD)-HSCT. MMF was given orally at 250 mg twice daily from day −9 to day +100. MTX was administered at a dose of 15 mg/m^2^ on day +1 and 10 mg/m^2^ on days +3, +6, +9, and +11. For Philadelphia chromosome (Ph)-positive patients, prophylactic maintenance therapy with a sensitive tyrosine kinase inhibitor (TKI) was administered 3 months after allo-HSCT and continued for at least 1 year, based on tolerance and mutation status post-HSCT.

### Clinical response and definitions

High-risk ALL was defined as meeting at least one of the following criteria: 1) white blood cell count >30 × 10^9^/L for B-cell precursor (BCP)-ALL, 2) pro-B-ALL, 3) ALL with Ph, 4) ALL with t(v;11q23) or KMT2A rearrangements, 5) ALL with complex karyotype (≥5 unrelated clonal abnormalities) or hypodiploid ALL (<44 chromosomes), and 6) failure to achieve CR after the first induction therapy (27, 28). Refractory ALL was defined as a failure to achieve CR at the end of induction. Relapse was defined as recurrence of >5% bone marrow blasts or the presence of extramedullary disease. Morphological CR was defined as the presence of less than 5% blasts in the bone marrow, with >1 × 10^9^/L neutrophils and >100 × 10^9^/L platelets in the peripheral blood without extramedullary disease. MRD-negative was defined as less than 10^−4^ assessed by flow cytometry of the bone marrow. An absolute neutrophil count (ANC) >0.5 × 10^9^/L on the first day of three consecutive days and platelet count >20 × 10^9^/L on the first day of seven consecutive days without transfusion support were defined as neutrophil and platelet engraftment, respectively. Acute GVHD (aGVHD) and chronic GVHD (cGVHD) were evaluated according to the National Institutes of Health consensus guidelines.

OS was measured as the time from the day of allo-HSCT to the last follow-up visit or death from any cause. LFS was defined as the time from haplo-HSCT to disease relapse/progression or death from any cause, whichever occurred first. Relapse was defined as disease relapse or disease progression after HSCT. Non-relapse mortality (NRM) was defined as death without relapse or disease progression after HSCT. Death without aGVHD and relapse were competing events for aGVHD, while death without cGVHD was a competing event for cGVHD. Patients who survived ≥100 days were analyzed for cGVHD.

### Statistical analysis

The final data cutoff for this study was 7 November 2021. Categorical variables between groups were compared using the two-sided Pearson’s chi-square or Fisher’s exact test, while continuous variables were compared using the non-parametric Mann–Whitney U test. The probabilities of OS and LFS were calculated by the Kaplan–Meier method. Cumulative incidence was used to estimate the incidence of GVHD, relapse rate, and NRM. Univariate analyses were conducted using Gray’s test for GVHD, relapse, and NRM, and the log-rank test for OS and LFS. All continuous variables were categorized, and the median was used as a cutoff point. The Fine–Gray proportional hazards regression model was used for multivariate analysis of GVHD, NRM, and relapse. The Cox proportional hazards regression model with stepwise forward selection was used for multivariate analysis of OS and LFS. To adjust for multiple testing of the pretransplant treatment status for OS and LFS outcomes, *p*-values were corrected using the Benjamini–Hochberg method after the log-rank test. Statistical significance was set at *p* < 0.05, and all *p*-values were two-sided. All statistical analyses were performed using R version 4.1.0 (R Core Team [2021]. R: A language and environment for statistical computing. R Foundation for Statistical Computing, Vienna, Austria. URL https://www.R-project.org/).

## Results

### Patient characteristics

**Figure 1:**
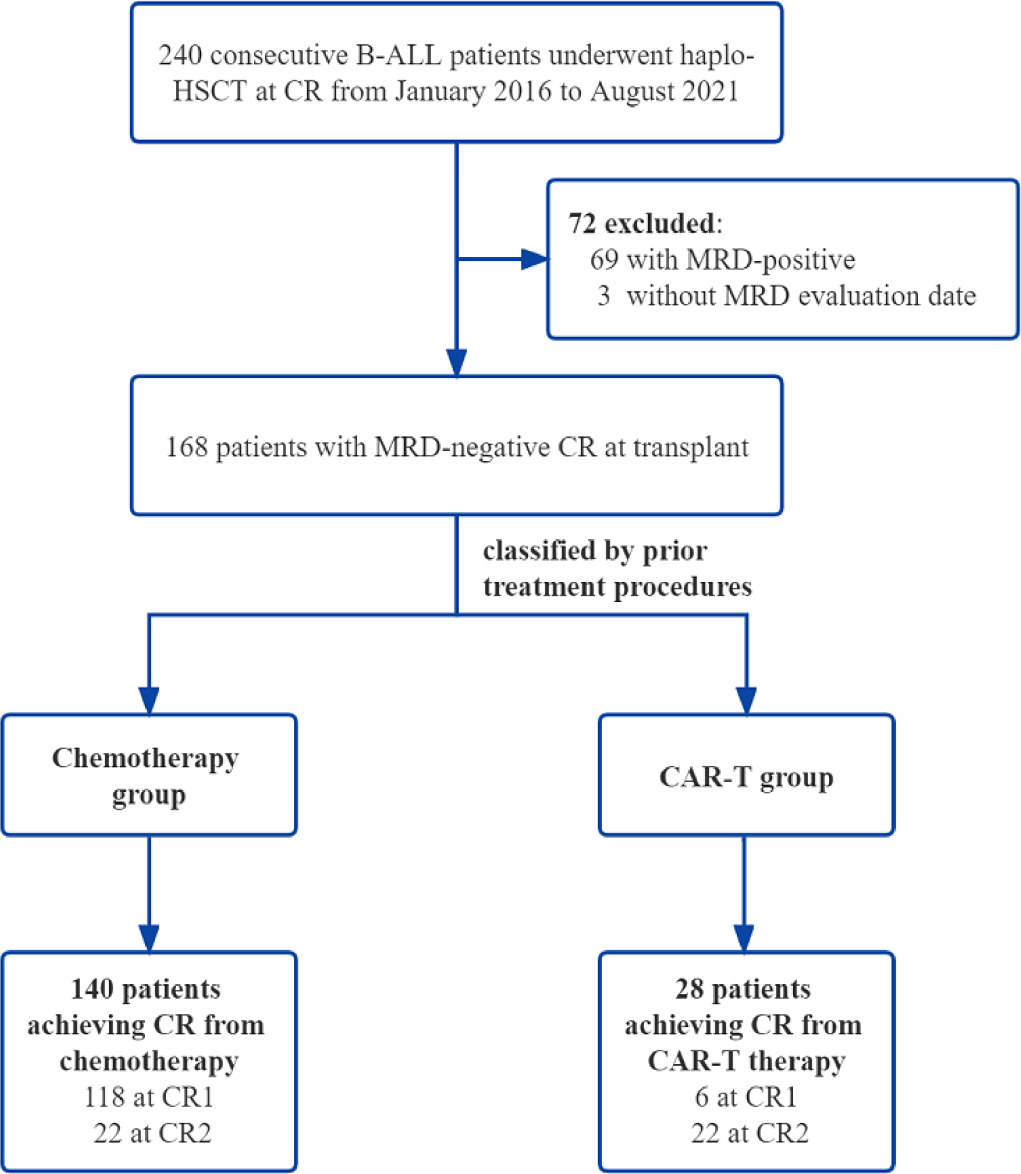
Patient enrollment flowchart.

Among the B-ALL patients admitted to our institute from 2016 to 2021, 168 patients with MRD**^−^** CR who underwent haplo-HSCT were enrolled (including two patients with a lymphoblastic crisis of chronic myelogenous leukemia) (**Figure 1**). The clinical characteristics of the two groups are summarized in **Table 1**. The median age of the entire cohort at diagnosis was 30.0 years (range, 7.4–62.3 years), and 78 patients (46.5%) were male. Fifty patients (30.2%) had BCR-ABL fusion gene with the treatment of tyrosine kinase inhibitors (**Supplementary Table 1**). The median time from diagnosis to haplo-HSCT was 7.0 months (range, 2.8–112.8 months). A total of 13 patients received DLI as preemptive therapy given the high risk of recurrence or R/R disease status: seven in the chemotherapy cohort (two in chemo+CR1 and five in the chemo+≥CR2) and six in the CAR-T cohort.

**Table 1:** Baseline and transplant-related characteristics of patients.

Characteristics	Total (N = 168)	Chemotherapy group (N = 140)	CAR-T group (N = 28)	*p*-Value
**Median age at diagnosis, years (range)**	30.0 (7.4–62.3)	29.5 (10.4–62.3)	31.4 (7.4–62.2)	0.333
**Gender, n (%)**				1.000
Male	78 (46.4)	65 (46.4)	13 (46.4)	
Female	90 (53.6)	75 (53.6)	15 (53.6)	
**Disease type**				1.000
B-ALL	166 (98.8)	138 (98.6)	28 (100.0)	
CML-LBP	2 (1.2)	2 (1.4)	0 (0.0)	
**Extramedullary disease involvement before HSCT**	13 (7.7)	12 (8.6)	1 (3.6)	0.605
**Fusion genes**				0.024
BCR-ABL1	50 (29.8)	47 (33.6)	3 (10.7)	
E2A-PBX1	4 (2.4)	4 (2.4)	0 (0.0)	
EVI1	2 (1.2)	1 (0.7)	1 (3.6)	
MLL-AF4	6 (3.6)	5 (3.6)	1 (3.6)	
TEL-AML	2 (1.2)	1 (0.7))	1 (3.6)	
MLL-ENL	1 (0.6)	0 (0.0)	1 (3.6)	
P2RY8-CRLF2	1 (0.6)	1 (0.7)	0 (0.0)	
**Poor cytogenetic risk, n (%)**	61 (36.3)	55 (39.3)	6 (21.4)	0.073
**Risk classification, n (%)**				0.110
Standard	58 (34.5)	52 (37.1)	6 (21.4)	
High	110 (65.5)	88 (62.9)	22 (78.6)	
**Relapsed/refractory, n (%)**	60 (35.7)	32 (22.9)	28 (100)	<0.001
**Prior HSCT**	3 (1.8)	2 (1.4)	1 (3.6)	0.423
**Median time from diagnosis to HSCT, months (range)**	7.0 (2.8–112.8)	6.8 (2.8–112.8)	8.7 (4.8–111.5)	0.002
**Median age at HSCT, years (range)**	30.7 (11.2–62.8)	30.7 (11.2–62.8)	29.6 (15.7–62.8)	0.416
**Disease status at HSCT**				<0.001
CR1	124 (73.8)	118 (84.3)	6 (21.4)	
≥CR2	44 (26.2)	22 (15.7)	22 (78.6)	
**Donor–patient gender, n (%)**				1.000
Female to male	18 (10.7)	15 (10.7)	3 (10.7)	
Others	150 (89.3)	125 (89.3)	25 (89.3)	
**Donor age, median (range)**	36.0 (11.0–60.0)	34.0 (11.0–60.0)	39.5 (15.0–60.0)	0.643
**ABO, n (%)**				0.298
Matched	93 (55.4)	80 (57.1)	13 (46.4)	
Mismatched	75 (44.6)	60 (42.9)	15 (53.6)	
**Conditioning regimen, n (%)**				0.871
Myeloablative	160 (95.2)	134 (95.7)	26 (92.9)	
RIC	8 (4.8)	6 (4.3)	2 (7.1)	
**MNC cell dose, ×10^8^/kg (range)**	13.1 (2–46)	13.1 (2–46)	13.0 (8–35)	0.988
**CD34+ cell dose, ×10^6^/kg (range)**	5.5 (1.5–17.9)	5.4 (1.5–17.9)	6.2 (2.5–15.4)	0.287
**Neutrophil engraftment, days (range)**	13 (8–29)	13 (8–24)	13.5 (10–29)	0.431
**Platelet engraftment, days (range)**	14 (8–35)	14 (8–35)	15 (9–29)	0.129
**Median follow-up of survivors,** **months (range)**	31.0 (3.6–70.1)	33.3 (3.6–70.1)	25.4 (4.3–65.8)	
**Preemptive therapy after HSCT**				
DLI	13 (7.7)	7 (5)	6 (21.4)	0.009

B-ALL, B-cell acute lymphoblastic leukemia; CAR-T, chimeric antigen receptor-T; CML-LBP, chronic myeloid leukemia-lymphoid blast phase; CR, complete remission; HSCT, hematopoietic stem cell transplantation; MNC, mononuclear cell; RIC, reduced intensity conditioning.

In the chemotherapy group, 32 patients (22.9%) had R/R B-ALL, three of whom developed two relapses. Two patients had a history of undergoing HSCT. In the CAR-T group, all 28 patients received 19-41-BB CAR T cells, and the median time from CAR-T infusion to haplo-HSCT was 2.65 months (range, 1.8–6 months). Five patients (17.9%) received CAR-T therapy for refractory ALL, one patient had persistent detectable MRD after CR1 in the bone marrow, and the remaining 22 patients (78.6%) had relapsed previously and received CAR-T therapy as salvage treatment. Among relapsed patients, the median time from diagnosis to first relapse was 5.3 months (range, 2.6–95.6 months). Following the first relapse, seven patients directly underwent CAR-T therapy; 11 failed to achieve CR after chemotherapy and received CAR-T (one patient successively received CAR-T twice because of relapse after the first CAR-T); three who had relapsed after consolidation treatment (two with regular chemotherapy and one with chemotherapy and subsequent MUD-HSCT) received CAR-T therapy directly and one patient for persistent detectable MRD after CR2 of chemotherapy. The details of salvage therapies in R/R patients are summarized in **Supplementary Table 2**. There were no significant differences in age, sex, or transplant characteristics between the two groups.

### Comparable outcomes between the chemotherapy group and the chimeric antigen receptor T-cell therapy group

**Figure 2:**
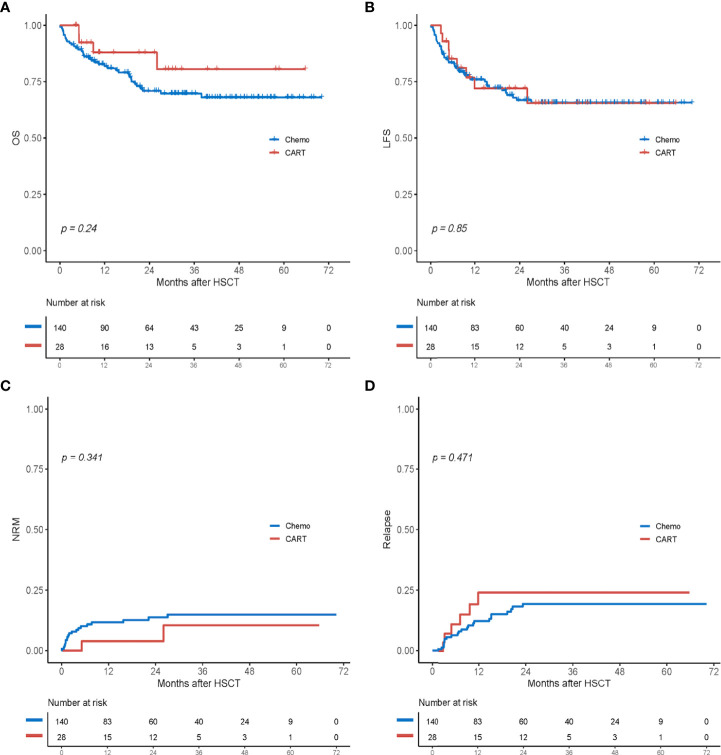
Outcomes after haploidentical HSCT between the chemotherapy group (chemo) and the CAR-T group. **(A)** OS. **(B)** LFS. **(C)** Cumulative incidence of non-relapse mortality (NRM). **(D)** Cumulative incidence of relapse. HSCT, hematopoietic stem cell transplantation; CAR-T, chimeric antigen receptor T cell; OS, overall survival; LFS, leukemia-free survival.

With a median follow-up period of 31.0 months among survivors (range, 3.6–70.1 months), 41 (24.4%) patients died (**Table 2**). In the chemotherapy cohort, 37 (26.4%) patients died, and the contributors to death were relapse (n = 16, 11.4%), infection (n = 12, 8.6%), severe GVHD (n = 3, 2.1%), organ failure (n = 3, 2.1%), thrombotic microangiopathy (n = 2, 1.4%), and secondary graft failure with infection (n = 1, 0.7%). By contrast, four (14.3%) patients died in the CAR-T group: two died of relapse at 9.0 and 5.1 months post-HSCT, one died of severe lung cGVHD with infection at 26 months, and one died of acute respiratory failure at 5.1 months after HSCT. The 2-year OS and LFS of patients in the chemotherapy group were 71.5 % and 66.8%, respectively, compared with 87.9% and 72.0% in the CAR-T group (*p* = 0.24 and *p* = 0.85; **Figures 2A, B**). At 2 years, the cumulative incidences of NRM in the chemotherapy and CAR-T groups were 13.7% and 3.9%, respectively (*p* = 0.341; **Figure 2C**).

**Table 2:** Causes of death.

Cause	All (n = 41)	Chemotherapy group (n = 37)	CAR-T group (n = 4)
Relapse	18	16	2
Infections	12	12	0
GVHD	4	3	1
Organ failure	4	3	1
TMA	2	2	0
Secondary graft failure	1	1	0

CAR-T, chimeric antigen receptor-T; GVHD, graft-versus-host disease; TMA, thrombotic microangiopathy.

A total of 29 patients (17.3%) experienced relapse post-HSCT: six (21.4%) in the CAR-T group and 23 (16.4%) in the chemotherapy group. In the chemotherapy group, 17 patients (12.1%) relapsed in the bone marrow, five (3.6%) relapsed in the bone marrow and had the presence of extramedullary disease, and one (0.7%) relapsed in the extramedullary group. In the CAR-T group, one patient relapsed in her eye 3.3 months after HSCT and then underwent enucleation. The other five patients relapsed in the bone marrow: two were lost to the last follow-up, two underwent second CAR-T therapy, and one was treated with chemotherapy plus donor lymphocyte infusion. The cumulative incidence of relapse at 2 years was 19.4% in the chemotherapy group and 24.1% in the CAR-T group (*p* = 0.471; **Figure 2D**).

### Multivariate analysis for transplant outcomes and graft-versus-host disease

**Figure 3:**
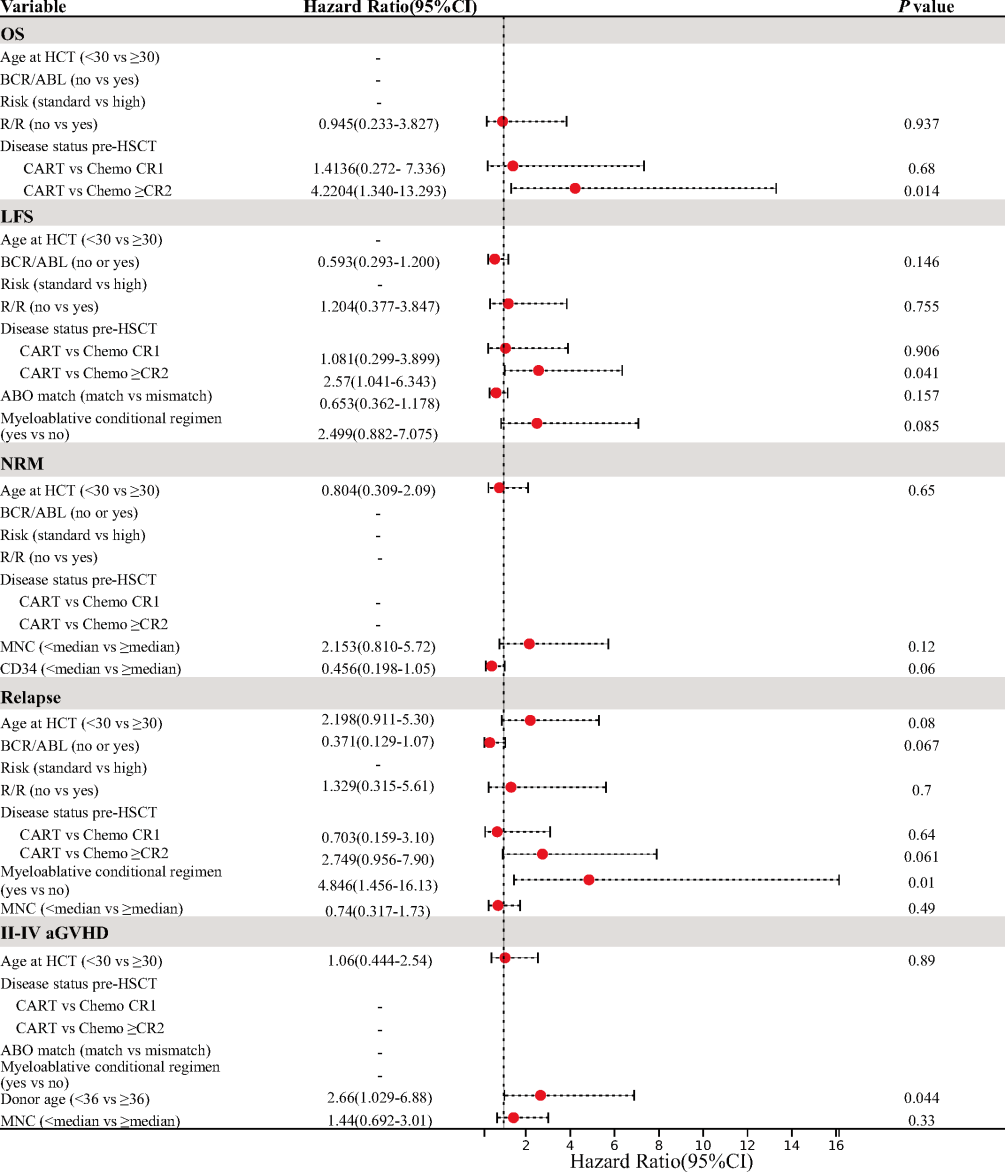
Multivariate analysis for OS, LFS, NRM, relapse, and II–IV aGVHD. OS, overall survival; LFS, leukemia-free survival; NRM, non-relapse mortality; aGVHD, acute graft-versus-host disease.

The results of the multivariate analysis are presented in **Figure 3**. In multivariate analysis of OS and LFS, patients undergoing HSCT performed in MRD-negative ≥CR2 post-chemotherapies had inferior OS and LFS compared to those undergoing post-CAR-T therapy (OS, hazard ratio (HR) 4.22 [95% CI, 1.34–13.293], *p* = 0.014; LFS, HR 2.57 [95% CI, 1.041–6.343], *p* = 0.041), but they did not show statistical significance for NRM and relapse in both univariate and multivariate analyses. The RIC conditioning regimen was an independent factor associated with a significantly higher risk of relapse (HR 4.846 [95% CI, 1.456–16.13], *p* = 0.01). Multivariate analysis for aGVHD indicated that donor age ≥36 years was a significant risk factor for the incidence of II–IV aGVHD (HR 2.66 [95% CI, 1.029–6.88], *p* = 0.044).

### Subgroup analysis among the chemo+CR1 group, the chemo+≥CR2, and the chimeric antigen receptor T-cell therapy group

**Figure 4:**
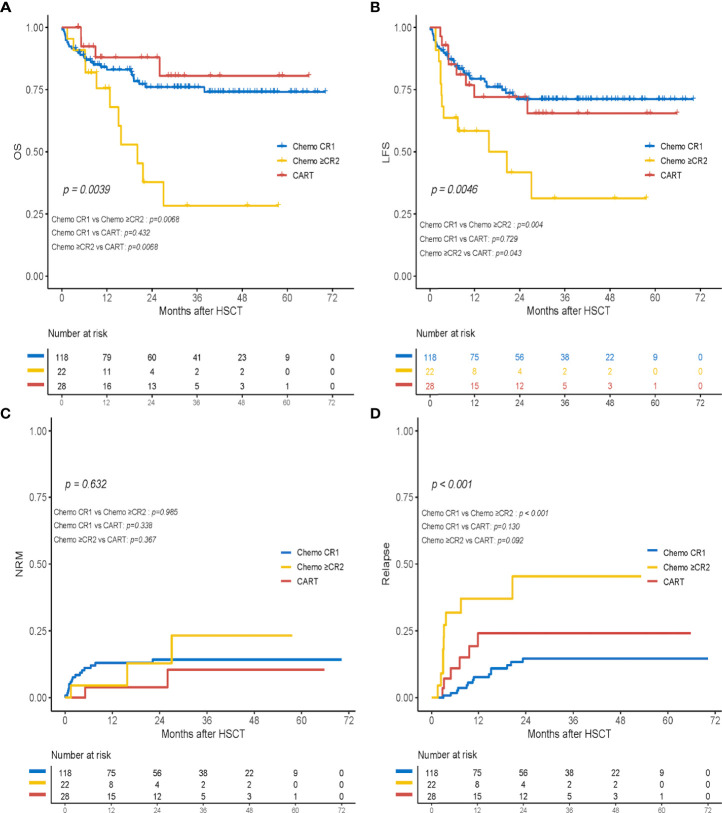
Transplant outcomes of subgroup analysis in the chemotherapy+CR1group (chemo+CR1), the chemotherapy+≥CR2 group (chemo+≥CR2) and the CART group. **(A)** OS; **(B)** LFS; **(C)** cumulative incidence of non-relapse mortality (NRM); and **(D)** cumulative incidence of relapse.

In the subgroup analysis, 11 patients (50%) in the chemo+≥CR2 cohort died: seven due to relapse and four due to infections after HSCT. The probabilities of OS and LFS at 2 years in the CAR-T group were comparable to those in the chemo+CR1 group (OS, 87.9% vs. 76.0%, *p* = 0.432; LFS, 72.0% vs. 71.1%, *p* = 0.729) but significantly higher than those in patients who received haplo-HSCT in MRD-negative ≥CR2 post-chemotherapy (OS, 87.9% vs. 37.8%, *p* = 0.007; LFS, 72.0% vs. 41.7%, *p* = 0.043) (**Figures 4A, B**). Further comparison of survival outcomes among the chemo+CR1, chemo+ ≥CR2, CAR-T CR1 (n = 6), and CAR-T+≥CR2 (n = 22) is provided in **Supplementary Figure 1**. The cumulative 2-year NRM incidence was 14.3%, 12.9%, and 3.9% in the chemo+CR1, chemo+≥CR2, and CAR-T cohorts, respectively. No significant differences were observed between the groups (**Figure 4C**). The cumulative incidences of relapse at 2 years were 14.6%, 45.5%, and 24.1% in the chemo+CR1, chemo+≥CR2, and CAR-T groups, respectively (**Figure 4D**). Patients in the chemo+CR1 group had a significantly lower relapse rate than those in the chemo+≥CR2 group (*p* < 0.001), but there was no difference from those in the CAR-T group (*p* = 0.130). There appeared to be a trend toward a higher relapse rate in the chemo+≥CR2 group than in the CAR-T group, but this difference was not statistically significant (*p* = 0.092).

### Engraftment and graft-versus-host disease after haploidentical hematopoietic stem cell transplantation

The median MNC cell dose was 13.1 × 10^8^/kg (range, 2–46 × 10^8^/kg), and the median CD34^+^ cell dose was 5.6 × 10^6^/kg (range, 1.5–15.4 × 10^6^/kg). The myeloablative conditional regimen was administered to 95.7% of patients in the chemotherapy group and 92.9% of patients in the CAR-T group. Among the patients surviving ≥30 days, all patients achieved neutrophil engraftment, and five developed primary platelet engraftment failure (four in the chemotherapy group and one in the CAR-T group). The median time of neutrophil engraftment was 13 days (range, 8–24 days) in the chemotherapy group and 13.5 days (range, 10–29 days) in the CAR-T group (*p* = 0.431), whereas the median time of platelet engraftment was 14 days (range, 8–35 days) in the chemotherapy cohort and 15 days (range, 9–29 days) in the CAR-T cohort (*p* = 0.129).

The cumulative incidence of grade I to IV aGVHD at 3 months was 33.6% in the chemotherapy group and 35.7% in the CAR-T group (*p* = 0.782; **Figure S2A**), and the cumulative incidence of grades II to IV aGVHD was 19.3% in the chemotherapy group and 17.9% in the CAR-T group (*p* = 0.842; **Figure S2B**). Of the 156 evaluable patients (92.9%), the incidence of chronic GVHD was not significantly different between the chemotherapy and CAR-T cohorts (32.1% vs. 38.6%, *p* = 0.918; **Figure S2C**). Subgroup analyses revealed that rates of I-IV aGVHD, II-IV aGVHD, and cGVHD were not significantly different among the three groups (*p* = 0.96, *p* = 0.921, and *p* = 0.359) (**Figures S2D–F**).

### Other complications after transplantation

The other transplant-associated complications are listed in **Supplementary Table 3**. In the chemotherapy group, 28 (20%) and 12 (8.6%) patients experienced at least one bacterial and invasive fungal infection, respectively. A total of 35 episodes of bacterial infections were documented, including 11 with *Klebsiella pneumoniae*, six with *Escherichia coli*, three with *Pseudomonas aeruginosa*, two with *Clostridium difficile*, two with *Staphylococcus haemolyticus*, and 11 with other bacteria. For the CAR-T group, the percentage of patients who had at least one bacterial and invasive fungal infection was 10.7% (3/28; one with *P. aeruginosa*, one with *K. pneumoniae*, and one with *S. aureus*) and 10.7% (3/28). The 2-year incidences of CMV and Epstein–Barr virus (EBV) infections were respectively 67.2% and 46.3% in the chemotherapy group and 75.5% and 58.4% in the CAR-T group. Thirty patients (24 in the chemotherapy group and six in the CAR-T group) had cystitis. Two patients in the chemotherapy group and one patient in the CAR-T group had immune thrombocytopenia and received a high dose of gamma globulin. EBV-associated post-transplant lymphoproliferative disorders occurred in three patients (two in the chemotherapy group and one in the CAR-T group), and all responded to rituximab. One patient in the chemotherapy group developed thrombotic thrombocytopenic purpura/hemolytic uremic syndrome (TTP/HUS).

## Discussion

Consolidative HSCT after CAR-T therapy has been recommended in some prospective studies to sustain long-term LFS (15, 18). However, few studies have compared transplant outcomes in patients after achieving CR either post-CAR-T or after chemotherapy, especially in the setting of deeper remission (MRD**^−^** CR) at the time of HSCT. In the current study, with a long-term follow-up of 31.0 months, we observed similar survival outcomes and relapse rates between the chemotherapy and CAR-T groups. Remission status ≥CR2 at transplantation following chemotherapy was an independent risk factor for OS in the multivariate analysis. Subgroup analyses were performed to evaluate the impact of the first remission at the time of allo-HSCT in patients undergoing transplantation. OS and LFS at 2 years in the CAR-T group were similar to those in the chemo+CR1 group but significantly higher than those in the chemo+≥CR2 group. To the best of our knowledge, this is the first study to compare the clinical prognoses of patients undergoing haplo-HSCT after achieving MRD**^−^** CR post-CAR-T therapy and post-chemotherapy.

Our data indicated that R/R patients who underwent HSCT after CAR-T therapy had comparable outcomes with the chemotherapy group, with 2-year OS, LFS, relapse, and NRM of 87.9%, 72.0%, 24.1%, and 3.9%, respectively. This result largely benefited from pretransplant deep remission induced by CAR-T therapy. Patients who reach MRD**^−^** CR at the time of HSCT have improved allo-HSCT outcomes (5–9, 29). Hu et al. reported that patients with MRD-negative post-CAR-T therapy had significantly better transplant outcomes than those who were MRD-positive (2-year OS of 89.8% vs. 63.6%, respectively) (29). In addition, results from our previous study analyzing the outcomes of R/R B-ALL patients who underwent haplo-HSCT after CAR-T therapy demonstrated improved LFS at 2 years in patients with MRD**^−^** CR than in patients with MRD-positive CR (76.1% vs. 27.6%, *p* = 0.007) (30). Furthermore, previous studies illustrated the superiority of CD19 CAR-T over chemotherapy in inducing the molecular response of ALL, with an MRD**^−^** CR rate of 60%–90%. An analysis of Peking University People’s Hospital found a higher rate of MRD^−^CR1 in the CAR-T group than in the chemotherapy group (90.7% vs. 70.5%, *p* = 0.036), and patients who received allo-HSCT after CAR-T therapy had a better 3-year LFS than patients after chemotherapy (77.8% vs. 68.7%, *p* = 0.575) (31). In addition, a significant achievement of MRD**^−^** CR after CAR-T therapy was observed in Ph+ ALL R/R patients with poor prognosis, with an MRD^−^CR rate of 67.9% (32). Thus, for patients with persistent MRD positivity or fusion gene positivity, CAR-T therapy instead of chemotherapy should be preferred as a bridge to allo-HSCT, decreasing persistent MRD pre-HSCT.

The optimal role of allo-HSCT in adults with ALL in CR1 needs to be revisited in the current era of effective immunotherapy agents, especially in MRD-negative remission (33–37). Although several prospective studies have demonstrated similar long-term outcomes between MRD**^−^** CR1 patients with allo-HSCT and without allo-HSCT (37–39), some patients with MRD**^−^** CR1 choose to undergo allo-HSCT because of the inability to achieve CR2 in cases of relapse and poor prognosis after transplantation in CR2. However, the advancement of CAR-T therapy has transformed the landscape of R/R ALL in recent years and is beginning to mark the frontline. Through CAR-T treatment, relapsed patients have more opportunities to achieve deep remission again and then proceed to transplant in CR2. The long-term prognosis of allo-HSCT performed in MRD-CR after CAR-T therapy remains unelucidated. Our results suggest that R/R patients undergoing allo-HSCT in deep CR induced by CAR-T therapy achieved outcomes similar to those of patients transplanted in MRD**^−^** CR1 after chemotherapy. This indicates that relatively favorable outcomes may be achieved when allo-HSCT is performed in patients with MRD**^−^** CR2 after CAR-T therapy, even if leukemia relapse occurs in patients without allo-HSCT in MRD**^−^** CR1. The need for allo-HSCT in patients with MRD**^−^** CR1 may decrease as the efficacy of CAR-T therapy increases. Our observations provide useful guidance for the administration of CAR-T therapy and the timing of allo-HSCT in ALL treatments.

Another notable finding in our study was the improved OS and LFS in R/R patients who received haplo-HSCT after achieving CR from CAR-T than that in relapsed patients after achieving CR2 or later from chemotherapy, despite the achievement of molecular remission in both groups. In the chemo+≥CR2 group, the incidence of 2-year relapse was 45.5%, which was significantly higher than those of the other two groups. Relapse was the most common cause of death, and NRM at 2 years was 12.9%. Conversely, among the CAR-T group, the 2-year incidences of relapse and NRM were only 24.1% and 3.9%, respectively, and improved OS and LFS at 2 years were observed. For ALL patients with first relapse, the choice of salvage treatment strategy may be more critical when allo-HSCT is performed during CR2 instead of CR1, considering the relatively inferior outcomes in patients transplanted in CR2. Our study demonstrated that CAR-T therapy should be preferred as salvage treatment, before allo-HSCT, for first relapsed patients, rather than chemotherapy, after chemotherapy, or for refractory patients, which could achieve transplant outcomes comparable to those in patients transplanted in CR1 in the context of deep remission.

The immunosuppressive and immunomodulatory effects of CAR-T therapy might alter the safety profile of subsequent allo-HSCT (40, 41). Few studies have compared transplant outcomes between patients with CR after either CAR-T therapy or post-chemotherapy. Recently, Zhao et al. conducted a parallel comparison of outcomes among patients with B-ALL who received allo-HSCT after achieving CR with CAR-T therapy (n = 27) or chemotherapy (n = 78). The results demonstrated that there were no significant differences in clinical outcomes (OS, LFS, NRM, relapse, and extensive cGVHD) between the two groups, while a higher incidence of II–IV aGVHD (48.1%) and slower platelet engraftment (14 days) were observed in the CAR-T group (42). In a previous study, Shadman et al. reported no increased risk of adverse events after HSCT with prior CAR-T therapy (43). Moreover, no correlation between the incidence and/or severity of CRS and the subsequent incidence and/or severity of acute GVHD has been observed in CAR-T patients (24). Likewise, although all patients in our cohort received haplo-HSCT, the incidence of GVHD was not very high and similar in both groups. Multivariable analyses showed that younger age in donors was a protective factor for II–IV aGVHD. In addition, our results showed that CAR-T therapy did not increase the risk of post-transplant infection. The incidence of bacterial and fungal infections was low in both groups, and the overall rates of viral infection were also comparable between the two groups. Consequently, CAR-T therapy followed by allo-HSCT is viable and safe for R/R patients and does not increase transplant-related mortality and toxicity.

There are some limitations to the present study, such as its retrospective design and the single-center design, each of which may have influenced the statistical results. Although CAR-T therapy had a higher remission response in R/R patients than conventional chemotherapy, the sample size of the CAR-T group in our study was relatively small, which is probably due to the expensive fee and strict enrollment criteria. The imbalanced sample size of the two groups may cause a potential bias. Although all patients achieved MRD**^−^** CR at the time of haplo-HSCT, a minority of patients still have positive mutated or fusion genes. This cohort is too limited to allow the inference of definitive conclusions on the role of CAR-T therapy in comparison with chemotherapy in this population. Additionally, in our study, the threshold for MRD detected by flow cytometry is 10^4^. It is not confirmed that patients in these two cohorts achieve the same depth of remission, as patients with an MRD level of 10^5^ to 10^6^ have a greater risk of relapse. Multicenter, randomized, prospective studies are required to confirm our conclusions.

In conclusion, by inducing MRD**^−^** CR, CAR-T therapy is a safe and feasible bridging regimen for haplo-HSCT without an increased risk of transplanted-related mortality and GVHD. Moreover, CAR-T therapy may play a synergistic role with allo-HSCT in improving survival outcomes in relapsed B-ALL. Patients with a first relapse after conventional chemotherapy could prioritize CAR-T therapy as salvage treatment and then proceed to allo-HSCT following MRD**^−^** CR.

## Data availability statement

The raw data supporting the conclusions of this article will be made available by the authors, without undue reservation.

## Ethics statement 

The studies involving human participants were reviewed and approved by the Institutional Review Board of the First Affiliated Hospital, School of Medicine, Zhejiang University. Written informed consent to participate in this study was provided by the participants’ legal guardian/next of kin.

## Author contributions

T-TY and Y-XH designed the study and wrote the paper. YM conducted the data analysis. D-LK, H-LZ, M-YZ, and R-RJ participated in data collection. H-LZ and M-MZ participated in the data analysis. G-QW, W-JW, J-MS, YL, Y-MZ, and JY took care of the patients and contributed to the clinical protocol. HH and Y-XH had the final responsibility to submit the paper for publication. All the authors reviewed and approved the paper.

## Funding

This work was funded by the National Natural Science Foundation of China (grant nos. 82130003 and 81870153) and Zhejiang Key R&D Program (Science and Technology Department, 2020C03G2013586).

## Acknowledgments

The authors thank all the doctors at the institute who participated in this study by providing the follow-up samples and information.

## Conflict of interest

The authors declare that the research was conducted in the absence of any commercial or financial relationships that could be construed as a potential conflict of interest.

## Publisher’s note

All claims expressed in this article are solely those of the authors and do not necessarily represent those of their affiliated organizations, or those of the publisher, the editors and the reviewers. Any product that may be evaluated in this article, or claim that may be made by its manufacturer, is not guaranteed or endorsed by the publisher.

## Supplementary material

Supplementary Figure 1 Survival outcomes among the chemotherapy+CR1group (chemo+CR1), chemotherapy+≥CR2 group (chemo+≥CR2), CART+CR1and CART+≥CR2 group.

Supplementary Figure 2 Cumulative incidences of aGVHD and cGVHD. I-IV aGVHD **(A)**, II-IV aGVHD**(B)** and cGVHD **(C)** between the chemotherapy group (chemo) and the CART group. I-IV aGVHD **(D)**, II-IV aGVHD**(E)** and cGVHD **(F)** in the chemotherapy+CR1group (chemo+CR1), the chemotherapy+≥CR2 group (chemo+≥CR2) and the CART group.
