# Supplementary figures and images for: Comparable outcomes in patients with B-cell acute lymphoblastic leukemia receiving haploidentical hematopoietic stem cell transplantation: Pretransplant minimal residual disease-negative complete remission following chimeric antigen receptor T-cell therapy versus chemotherapy
Source: Front Immunol. 2022 Aug 30;13:934442. doi: 10.3389/fimmu.2022.934442 (PMC9468760; doi:10.3389/fimmu.2022.934442)

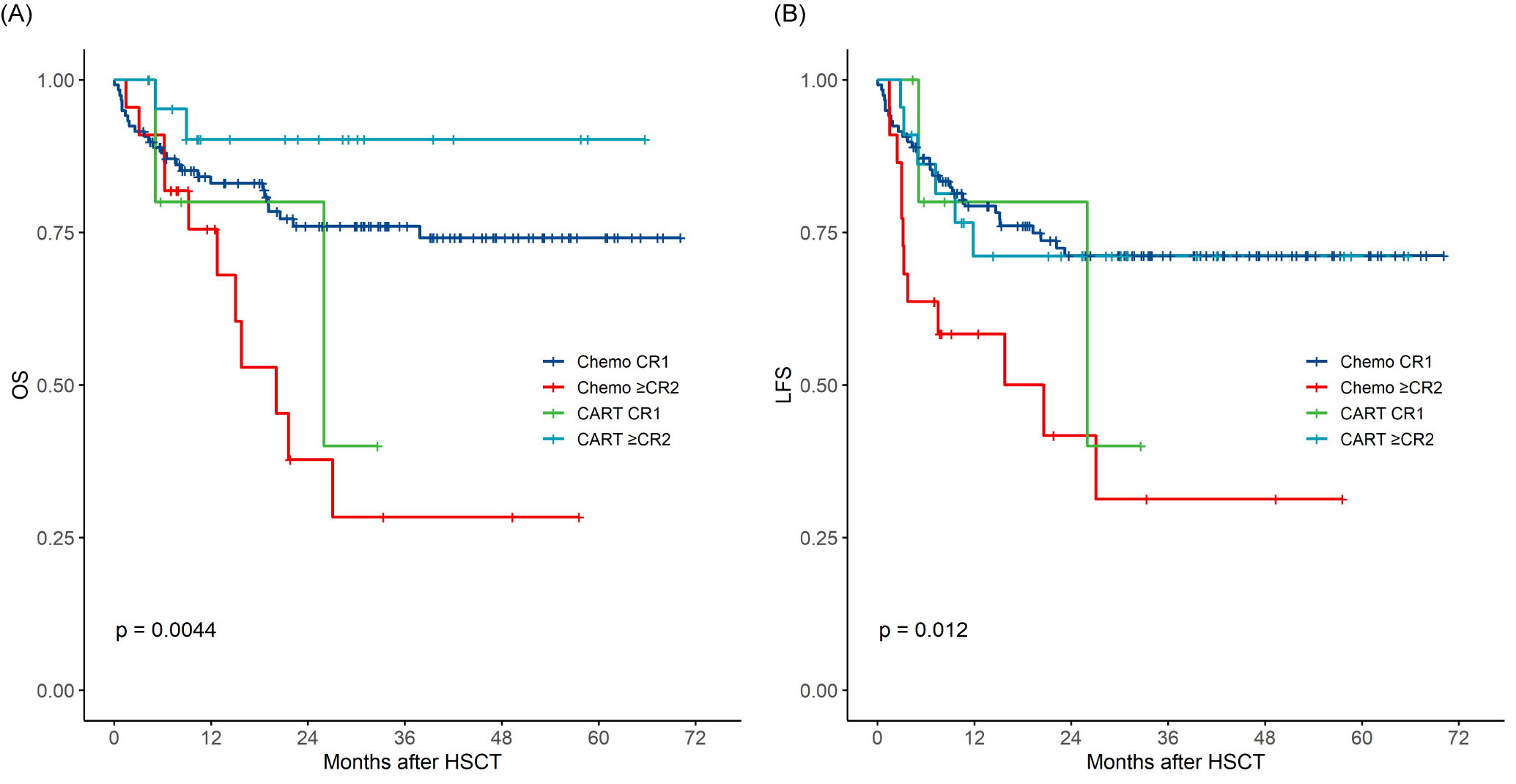

Supplement: Supplementary Figure 1 — Survival outcomes among the chemotherapy+CR1group (chemo+CR1), chemotherapy+≥CR2 group (chemo+≥CR2), CART+CR1and CART+≥CR2 group. [file Image_1.jpeg]

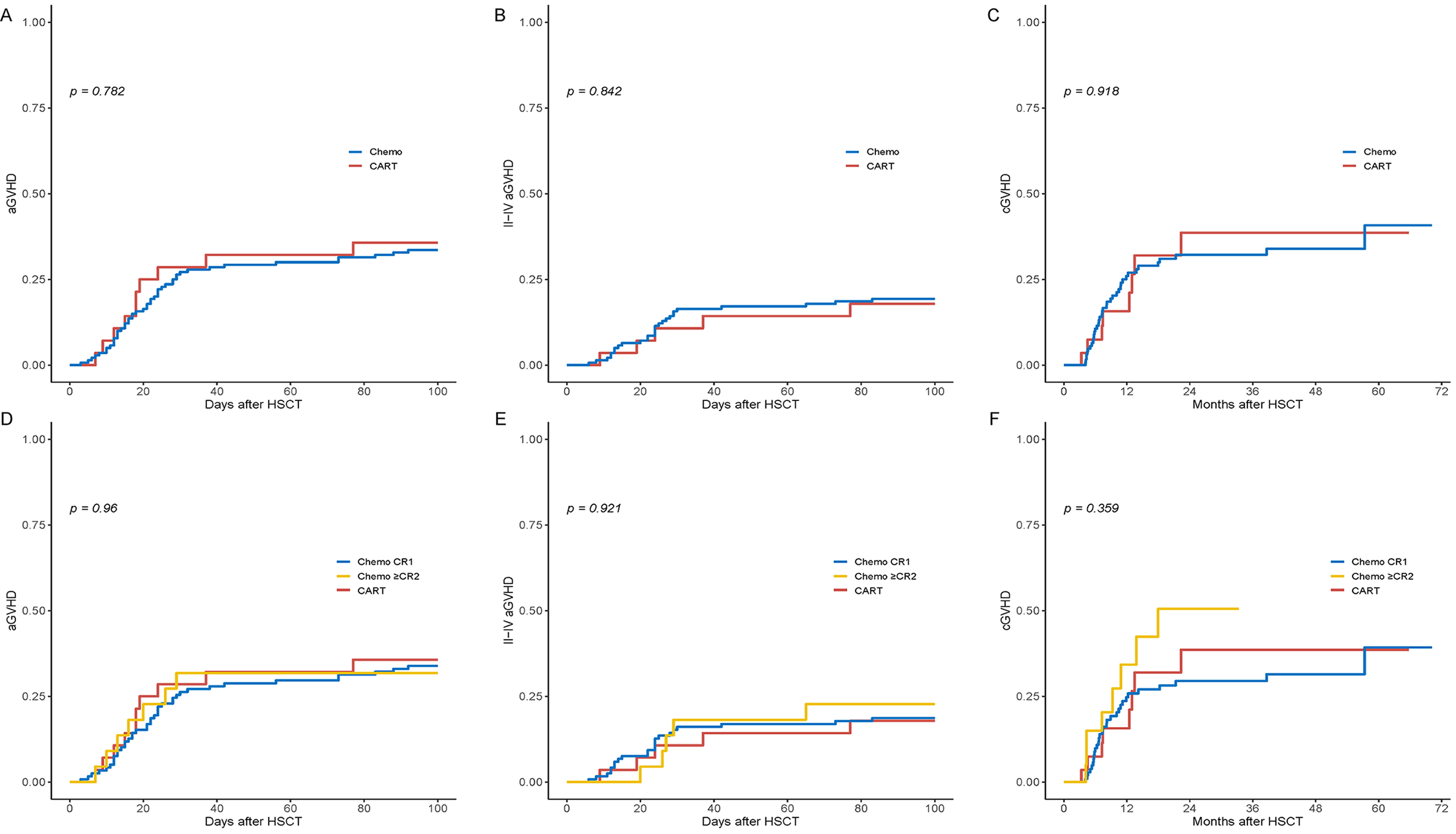

Supplement: Supplementary Figure 2 — Cumulative incidences of aGVHD and cGVHD. I-IV aGVHD (A), II-IV aGVHD(B) and cGVHD (C) between the chemotherapy group (chemo) and the CART group. I-IV aGVHD (D), II-IV aGVHD(E) and cGVHD (F) in the chemotherapy+CR1group (chemo+CR1), the chemotherapy+≥CR2 group (chemo+≥CR2) and the CART group. [file Image_2.tif]
