# Supplementary material for: Comparable outcomes in patients with B-cell acute lymphoblastic leukemia receiving haploidentical hematopoietic stem cell transplantation: Pretransplant minimal residual disease-negative complete remission following chimeric antigen receptor T-cell therapy versus chemotherapy
Source: Front Immunol. 2022 Aug 30;13:934442. doi: 10.3389/fimmu.2022.934442 (PMC9468760; doi:10.3389/fimmu.2022.934442)
Supplement: Supplementary file 3 [file Table_1.docx]

Supplementary Table 1 Administration of tyrosine kinase inhibitor pre-transplant among Philadelphia chromosome–positive patients

| **Tyrosine kinase inhibitor** | **Patients (n)** |
| --- | --- |
| Imatinib | 24 |
| Dasatinib | 18^#^ |
| Flumatinib | 1 |
| Nilotinib | 2^*^ |
| Ponatinib | 5^a^ |

Abbreviations：^#^One patient changed imatinib to dasatinib after disease relapse. ^*^Following disease relapse, one patient was discontinued with flumatinib and switched to nilotinib. ^a^A total of five patient switched Imatinib/dasatinib to ponatinib due to the refractory/relapsed disease.
