# Supplementary material for: Comparable outcomes in patients with B-cell acute lymphoblastic leukemia receiving haploidentical hematopoietic stem cell transplantation: Pretransplant minimal residual disease-negative complete remission following chimeric antigen receptor T-cell therapy versus chemotherapy
Source: Front Immunol. 2022 Aug 30;13:934442. doi: 10.3389/fimmu.2022.934442 (PMC9468760; doi:10.3389/fimmu.2022.934442)
Supplement: Supplementary file 4 [file Table_2.docx]

Supplementary Table 2 Pre-transplant salvage regimens among refractory/relapsed patients

|  | Chemotherapy group  (n=32) | CART group  (n=28) |
| --- | --- | --- |
| Chemotherapy* | 26 | 26 |
| Blinatumomab | 1 | 0 |
| Venetoclax combination therapy | 3 | 1 |
| Chidamide plus chemotherapy | 1 | 0 |
| DLI plus chemotherapy | 1 | 0 |
| Midostaurin plus chemotherapy | 0 | 1 |

*For Philadelphia chromosome–positive patients, TKI was added and was selected based on the gene mutation and diseases status.
