# Supplementary material for: Comparable outcomes in patients with B-cell acute lymphoblastic leukemia receiving haploidentical hematopoietic stem cell transplantation: Pretransplant minimal residual disease-negative complete remission following chimeric antigen receptor T-cell therapy versus chemotherapy
Source: Front Immunol. 2022 Aug 30;13:934442. doi: 10.3389/fimmu.2022.934442 (PMC9468760; doi:10.3389/fimmu.2022.934442)
Supplement: Supplementary file 5 [file Table_3.docx]

Supplementary Table 3. Transplant-associated complications

|  | **All**  **(N=168)** | **Chemotherapy group(N=140)** | **CAR-T group**  **(N=28)** |
| --- | --- | --- | --- |
| **Bacterial and fungal infection confirmed by culture** | 42(25.0) | 38(27.1) | 4(14.3) |
| **Viral infection** |  |  |  |
| CMV | 116(69.0) | 95(67.9) | 21(75.0) |
| EBV | 81(48.2) | 65(46.4) | 16(57.1) |
| **Other virus** |  |  |  |
| BK | 82(48.8) | 65(46.4) | 17(60.7) |
| JC | 39(23.2) | 30(21.4) | 9(32.1) |
| Parvovirus | 19(11.3) | 16(11.4) | 3(10.7) |
| Influenza virus | 3(1.8) | 2(1.4) | 1(3.6) |
| HHV-6 | 1(0.6) | 1(0.7) | 0 |
| **Cystitis** | 30(17.9) | 24(17.1) | 6(21.4) |
| **EBV associated PTLD** | 3(1.8) | 2(1.4) | 1(3.6) |
| **Primary platelet dysfunction** | 5(3.0) | 4(2.9) | 1(3.6) |
| **Toxoplasma encephalitis** | 1(0.6) | 1(0.7) | 0 |
| **ITP** | 3(1.8) | 2(1.4) | 1(3.6) |
| **TTP/HUS** | 1(0.6) | 1(0.7) | 0 |
| **Secondary malignancy** | 1(0.6) | 1(0.7) * | 0 |

ITP, Immune thrombocytopenia; TTP/HUS, thrombotic thrombocytopenic purpura/hemolytic uremic syndrome.

*The patient developed tongue cancer post-transplant.
